# 老年晚期NSCLC患者PD-1/PD-L1免疫检查点抑制剂治疗现状及展望

**DOI:** 10.3779/j.issn.1009-3419.2024.106.10

**Published:** 2024-05-20

**Authors:** MAO Yunye, SHENG Shu, WANG An, ZHAI Jinzhao, GE Xiangwei, LU Di, WANG Jinliang

**Affiliations:** ^1^100071 北京，解放军总医院第五医学中心肿瘤医学部肿瘤内科（卯云烨，盛舒，王安，翟今朝，葛祥伟，卢迪，汪进良）; ^1^Department of Oncology, Senior Department of Oncology, the Fifth Medical Center of PLA General Hospital, Beijing 100071, China; ^2^100853 北京，解放军医学院（卯云烨，盛舒，王安，翟今朝，葛祥伟，卢迪）; ^2^Chinese PLA Medical School, Beijing 100853, China

**Keywords:** 肺肿瘤, 老年非小细胞肺癌, 免疫衰老, 免疫治疗, 免疫联合低强度化疗, Lung neoplasms, Elderly non-small cell lung cancer, Immunosenescence, Immunotherapy, Immunotherapy combined with low-intensity chemotherapy

## Abstract

癌症发病率与年龄密切相关，75%的非小细胞肺癌（non-small cell lung cancer, NSCLC）患者年龄均≥65岁。免疫检查点抑制剂（immune checkpoint inhibitors, ICIs）的出现改变了NSCLC的治疗格局。针对老年患者有限的研究发现对于65-75岁的患者，ICIs单药显示出良好的获益，与年轻患者无明显差异，这种获益在免疫联合化疗或放疗中也有所体现。但是对于≥75岁的患者来说生存获益不明显。ICIs单药在不同年龄段的患者中免疫相关不良反应（immune-related adverse events, irAEs）发生率相似，免疫联合化疗对比单纯化疗导致irAEs的发生率高，≥75岁患者发生更高级别irAEs的可能性更大。除了老年患者免疫衰老会从多维度影响免疫微环境从而影响免疫治疗疗效外，东部肿瘤协作组体力状态（Eastern Cooperative Oncology Group performance status, ECOG PS）评分等也会影响预后。对于部分≥75岁或身体状况较差的患者，免疫联合低强度化疗成为有潜力的治疗方式之一，但相关研究较少，所以应有意识增加老年患者入组试验的人数，同时综合评估，探索个体化治疗方案。本综述拟对老年NSCLC患者应用抗程序性死亡受体1（programmed cell death protein 1, PD-1）及其配体（programmed cell death ligand 1, PD-L1）的相关研究进行汇总分析，以期为老年NSCLC患者的免疫治疗提供更多参考和指导，并结合其特点提出新的治疗展望。

## Abstract (en)

The incidence of cancer is closely correlated with age, as 75% of non-small cell lung cancer (NSCLC) patients are aged at least 65 years. The availability of immune checkpoint inhibitors (ICIs) has altered the available NSCLC therapeutic pattern. Limited studies on elderly patients have demonstrated that ICIs as monotherapy provide substantial benefits for patients aged 65-75 years, showing no significant difference compared to younger patients. This benefit is also observed in combination with immune-combined chemotherapy or radiotherapy. For individuals older than 75 years, the survival effect was not evident, though. Immune-related adverse events (irAEs) with ICIs alone were similar in incidence across age categories. Immune-combination chemotherapy resulted in a higher incidence of irAEs than chemotherapy alone, and patients ≥75 years of age were more likely to experience higher-grade irAEs. Besides the fact that immunosenescence in older patients influences the immune milieu in a multifaceted manner, which in turn impacts the effectiveness of immunotherapy, the prognosis is also influenced by the Eastern Cooperative Oncology Group performance status (ECOG PS) score, among other factors. For certain individuals aged ≥75 years or in poor physical health, immunotherapy combined with low-intensity chemotherapy has emerged as a viable treatment option. However, there are fewer related studies, so there should be a conscious effort to increase the number of elderly patients enrolled in the trial and a comprehensive assessment to explore individualized treatment options. To provide additional references and guidance for immunotherapy in elderly NSCLC patients and to propose new therapeutic perspectives in combination with their characteristics, this review aims to summarize and analyze the pertinent studies on the application of programmed cell death protein 1 (PD-1)/programmed cell death ligand 1 (PD-L1) inhibitors in these patients.

随着老龄化程度的加深，老年癌症患者将持续上升。2022年全球癌症数据^[1]^表明，约2/3以上的癌症患者年龄在65岁以上。国内数据^[2]^表明，60-79岁组及80岁以上组，肺癌居男、女癌症发病和死亡首位。非小细胞肺癌（non-small cell lung cancer, NSCLC）占所有肺癌的80%以上，大多数早期肺癌患者常无症状，确诊时已是晚期，5年生存率低于15%^[1]^。随着免疫治疗的发展，以抗程序性死亡受体1（programmed cell death protein 1, PD-1）及其配体（programmed cell death ligand 1, PD-L1）的抗体为代表的免疫检查点抑制剂（immune checkpoint inhibitors, ICIs）已成为晚期肺癌患者一线治疗的重要选择^[3]^。ICIs在晚期NSCLC领域得到广泛应用并使患者获益，5年生存率上升至15.5%-23.2%^[4]^。然而，由于老年患者可能存在复杂的合并症，免疫系统的衰老使老年癌症患者发生严重免疫相关不良反应（immune-related adverse events, irAEs）的风险增加，加之临床试验中老年患者占比不高，证据经验相对不足，使得这一人群的治疗面临巨大的未满足的需求。本文就老年NSCLC患者的免疫特征及现有ICIs治疗的效果与安全性数据进行综述，并展望免疫治疗对这一人群的获益前景。

## 1 老年NSCLC患者与免疫衰老

老年患者具有免疫衰老的特点，它是与年龄相关的免疫系统功能的失调和衰退，随着年龄的增长，免疫系统会逐渐衰老，即免疫衰老，它是人体衰老过程或现象在免疫系统中的表现，使人体免疫系统无法有效地识别、攻击和消灭外来病原体，主要表现在免疫器官的退化以及先天性和适应性免疫功能的障碍。首先，骨髓中的造血干细胞（haematopoietic stem cell, HSC）分化能力减弱，会分化成功能失调的免疫细胞；胸腺会逐渐萎缩，生成T细胞的能力减弱；淋巴结组织重塑，生发中心减少^[5]^。其次，免疫细胞亚群也会发生变化，先天性免疫细胞如树突状细胞（dendritic cells, DCs）对抗原的摄取减少，加工和呈递抗原的能力也下降；巨噬细胞更趋于向M2型转化，分泌免疫抑制因子；自然杀伤（natural killer, NK）细胞数量增加，CD56^bright^NK细胞比例降低，细胞因子和趋化因子的产生减少，穿孔素和颗粒酶的释放减少；髓源性抑制细胞（myeloid-derived suppressor cell, MDSC）数量增多；中性粒细胞迁移功能异常等，对刺激的反应降低，导致老年人对疾病的易感性增加。适应性免疫细胞如B细胞多样性减少，功能降低；T细胞数量减少，T细胞受体（T cell receptor, TCR）多样性丧失，CD28受体表达减少，调节性T细胞（regulatory T cells, Treg）、记忆T细胞数量增加等，导致免疫细胞无法有效抑制肿瘤的生长和转移。此外，免疫衰老会导致炎症和促炎因子的积累，衰老细胞可以产生血管生成因子、趋化因子、细胞因子等衰老相关分泌表型（senescence-associated secretory phenotype, SASP），从而形成炎性衰老，降低免疫反应效率，形成免疫抑制微环境^[6]^（表1）。综上，免疫衰老导致免疫细胞活化及杀伤能力降低，炎症衰老会形成慢性的炎症状态，肿瘤微环境招募免疫抑制细胞，进而抑制正常免疫细胞活动。虽然ICIs可以重新激活免疫细胞使其获得免疫功能，但是免疫衰老还是会对ICIs的疗效产生影响^[13]^。

**表 1:** 免疫衰老的特点

Immunosenescence characteristics	Region	Specific changes
Alterations in lymphoid organs	Bone marrow	The increased amount of hematopoietic tissue with a reduced regenerative capacity^[7]^ Multidirectional myeloid differentiation^[7]^ Changes in the microenvironment and increased deposition of adipose tissue^[7]^
	Thymus gland	Progressive regression of thymus size, deterioration of thymic stroma^[7]^
	Lymphatic node	Reduced size and function^[8]^
Alterations in cellular subpopulations	DC	Decrease in cell number^[9]^ Decreased TLR^[10]^ Decreased secretion of IFN-α^[9]^ Decreased phagocytosis and migration^[10]^
	B cell	Reduction in number and diversity^[6]^
	CD4^+ ^T cell	Reduced TCR diversity^[10]^ Decreased CD28 expression^[10]^ Reduced primary CD4^+ ^T cell activity and sensitivity^[10]^
	CD8^+ ^T cell	Reduced TCR diversity^[6]^ Decreased cell production^[6]^ Decreased CD28 expression^[6]^ Increased expression of CD57^[6]^ Increased expression of PD-L1^[6]^ Increased sensitivity to apoptotic signaling^[6]^ Decreased levels of perforin and granzyme^[11]^
	Tregs	Increased cell number and inhibitory activity^[9]^
	MDSCs	Increased numbers in tumor mesenchyme and circulation^[9]^ Expression of PD-L1 and inhibition of T cell activity^[12]^
	Macrophage	M2 macrophage increased^[6]^
	NK cell	CD56^dim^NK cell increased^[9]^
Alterations in the composition of circulating cytokines	Cytokine	Progressive increase in pro-inflammatory mediators, including TNF, IL-6 and IL-1^[6]^

DC: dendritic cell; Tregs: regulatory T cell; MDSCs: myeloid-derived suppressor cells; NK cell: natural killer cell; TLR: Toll-like receptor; IFN-α: interferon-α; PD-L1: programmed cell death ligand 1; TCR: T cell receptor; TNF: tumor necrosis factor; IL-6: interleukin-6; IL-1: interleukin-1.

## 2 老年晚期NSCLC的ICIs治疗效果

### 2.1 ICIs单药治疗

ICIs单药治疗在晚期NSCLC领域得到广泛应用，KEYNOTE-010^[14]^是首项评估帕博利珠单抗与多西他赛用于既往治疗过的PD-L1阳性的晚期NSCLC的疗效。研究发现免疫单药治疗使65-69岁患者的死亡风险降低24%。

KEYNOTE-024研究^[15]^是首个证实一线免疫单药治疗NSCLC优于化疗的试验，对于无表皮生长因子受体（epidermal growth factor receptor, EGFR）、间变性淋巴瘤激酶（anaplastic lymphoma kinase, ALK）突变且PD-L1表达≥50%的晚期NSCLC患者，帕博利珠单抗组比含铂双药化疗组具有更好的总生存期（overall survival, OS），亚组分析显示，在≥65岁人群中免疫单药组OS获益与整体人群一致，并显著优于化疗组[风险比（hazard ratio, HR）=0.64，95%CI：0.42-0.98]。KEYNOTE-042研究^[16]^将人群扩大到PD-L1表达水平在1%-49%的NSCLC患者中，发现≥65岁患者免疫组的OS优于化疗组（HR=0.82, 95%CI: 0.66-1.01）。

对以上3个试验中≥75岁且PD-L1表达阳性的患者汇总后，发现免疫单药组和化疗组的中位OS分别为15.7和11.7个月（HR=0.76, 95%CI: 0.56-1.02），PD-L1表达≥50%的患者中，免疫单药较化疗也显著改善OS，中位OS分别为23.1和8.3个月（HR=0.40, 95%CI: 0.25-0.64）^[17]^。然而，CheckMate 017、057和IMpower 110试验^[18⇓-20]^中免疫单药组对≥75岁患者获益不明确，而对65-75岁患者的OS有所获益或有获益趋势，与年轻患者一致。

老年患者除年龄因素的影响外，自身身体状况也是需要考虑的因素。CheckMate 171试验^[21]^补充了东部肿瘤协作组体力状态（Eastern Cooperative Oncology Group performance status, ECOG PS）评分≥2分且≥70岁NSCLC患者的临床数据。纳入患者在一线治疗失败后接受纳武利尤单抗治疗，所有接受治疗的患者、≥70岁和≥75岁的患者的中位OS相似，分别为10.0、10.0和11.2个月。

### 2.2 ICIs联合化疗

化疗可以通过多种机制激活免疫反应，因此免疫联合化疗是目前研究最多的联合治疗策略之一^[22]^。KEYNOTE-189试验^[23]^巩固了ICIs联合化疗在非鳞NSCLC患者一线治疗的地位。在亚组分析中观察到≥65岁的患者免疫联合化疗组和化疗组的无进展生存期（progression-free survival, PFS）分别为9.0和6.7个月（HR=0.75, 95%CI: 0.55-1.02）。针对鳞状NSCLC的试验KEYNOTE-407^[24]^中，与年轻患者相比，≥65岁患者PFS获益（HR=0.63, 95%CI: 0.47-0.84），但OS获益不明显。

IMpower 150^[25]^是首个也是唯一一个证实免疫联合抗血管生成治疗和化疗一线治疗非鳞状NSCLC的患者OS和PFS均获益的试验。意向治疗分析（intention-to-treat, ITT）人群中，65-74岁患者OS也显著获益（HR=0.76, 95%CI: 0.557-1.000），≥75岁的患者未观察到OS改善。

IMpower 131和IMpower 130研究^[26,27]^旨在探索转移性鳞状和非鳞NSCLC患者接受阿替利珠单抗联合化疗与单纯化疗的疗效差异，都显示出免疫联合化疗在OS和PFS方面的益处，但按年龄分层时免疫联合化疗组的OS获益无统计学意义。

一项大型回顾性研究^[28]^纳入4271例无EGFR或ALK突变的晚期NSCLC患者，一线采用不同的化疗和免疫联合治疗的方案。在鳞癌患者中，65-74岁的患者的中位OS比<65岁的患者或≥75岁的患者长，分别为14.5、8.9和9.3个月；在非鳞癌患者中，年龄越大中位OS越短，<65岁、65-74岁和≥75岁患者的中位OS分别为13.2、12.3和10.1个月。

### 2.3 双ICIs联合治疗

联合阻断PD-1/PD-L1与细胞毒性T淋巴细胞抗原-4（cytotoxic T lymphocyte antigen-4, CTLA-4）有协同作用^[29]^。CheckMate 227^[30]^是评估双免疫联合（纳武利尤单抗联合伊匹木单抗）疗法一线治疗晚期NSCLC的研究，无论PD-L1的表达情况如何，双ICIs治疗患者的OS显著延长，亚组中65-74岁患者的OS有所获益（HR=0.75, 95%CI: 0.61-0.92），≥75岁患者由于人数较少，在该研究中没有受益。

双免疫疗法中部分患者出现早期进展，为克服该问题，CheckMate 9LA研究^[31]^探索了双免疫联合有限化疗的疗效。研究发现使用双免疫联合化疗的患者中，<65岁、65-74岁和≥75岁的患者的中位OS分别为15.6、19.4和8.5个月，<65岁和65-74岁的患者中位OS获益明显，但≥75岁患者未见获益。

综上，汇总老年晚期NSCLC免疫治疗的试验发现（表2），“老年”不是免疫治疗的绝对禁忌，对于65-74岁的患者，使用ICIs单药治疗可以显著改善生存情况，尤其是对于PD-L1表达高的患者，且与年轻患者无明显差异，这种获益在免疫单药联合化疗、双免疫治疗或双免疫联合化疗的试验中也有所体现。但是对于≥75岁的患者，ICIs治疗的生存获益不明显，可能是由于老年患者样本量不足导致结果的代表性不充分，免疫衰老、身体状况较差导致疗效受限等，仍需要积累更多的临床数据，为优化这一人群的免疫治疗方案提供科学依据。

**表 2:** 晚期NSCLC免疫治疗随机临床试验中按年龄划分的OS或PFS

NSCLC trials	Type of experiment/ Line(s)	Recruit	Treatment pattern	Study endpoint	Sample and age distribution	Outcomes [HR (95%CI)]
KEYNOTE- 010^[14]^	ll/lll Second or further line	Previously treated PD-L1 TPS≥1%	Pembrolizumab vs Docetaxel	Primary: OS, PFS Secondary: ORR, DOR Safety	n=1034 Median age 63 years ≥65 years: 41%	OS ≥65 years: 0.76 (0.57-1.02) OS <65 years: 0.63 (0.50-0.79)
KEYNOTE- 024^[15]^	lll PD-L1≥50% First line	Untreated PD-L1 TPS≥50% EGFR/ALK negative	Pembrolizumab vs Platinum-based chemotherapy	Primary: PFS Secondary: OS, ORR Safety	n=305 Median age 65 years ≥65 years: 54%	OS ≥65 years: 0.64 (0.42-0.98) OS <65 years: 0.60 (0.38-0.96)
KEYNOTE- 042^[16]^	lll PD-L1≥1% First line	Untreated PD-L1 TPS≥1% EGFR/ALK negative	Pembrolizumab vs Platinum-based chemotherapy	Primary: OS (PD-L1 TPS≥50%) Secondary: OS, PFS	n=1274 Median age 63 years ≥65 years: 45%	OS ≥65 years: 0.82 (0.66-1.01) OS <65 years: 0.81 (0.67-0.98)
CheckMate 017^[18]^	lll Second line	Previously treated squamous	Nivolumab vs Docetaxel	Primary: OS Secondary: PFS, ORR Safety	n=272 Median age 63 years ≥65 years: 44%	OS ≥75 years: 1.85 (0.76-4.51) OS 65-74 years: 0.56 (0.34-0.91) OS <65 years: 0.52 (0.35-0.75)
CheckMate 057^[19]^	lll Second or third line	Previously treated nonsquamous	Nivolumab vs Docetaxel	Primary: OS Secondary: PFS, ORR	n=582 Median age 62 years ≥65 years: 42%	OS ≥75 years: 0.90 (0.43-1.87) OS 65-74 years: 0.63 (0.45-0.89) OS <65 years: 0.81 (0.62-1.04)
KEYNOTE- 189^[23]^	lll First line	Untreated nonsquamous EGFR/ALK negative	Platinum-based chemotherapy+ Pembrolizumab or placebo	Primary: OS, PFS Secondary: ORR, DOR Safety	n=616 Median age 64 years ≥65 years: 49%	OS ≥65 years: 0.64 (0.43-0.95) OS <65 years: 0.43 (0.31-0.61)
KEYNOTE- 407^[24]^	lll First line	Untreated squamous	Platinum-based chemotherapy+ Pembrolizumab or placebo	Primary: OS, PFS Secondary: ORR, DOR Safety	n=559 Median age 65 years ≥65 years: 55%	OS ≥65 years: 0.74 (0.51-1.07) OS <65 years: 0.52 (0.34-0.80)
IMpower 150^[25]^	lll First line	Untreated nonsquamous	Platinum-based chemotherapy and Bevacizumab±Atezolizumab	Primary: OS, PFS Secondary: OS in ITT and PD-L1 subgroups	n=800 Median age 63 years ≥65 years: 45%	PFS ≥75 years: 0.78 (NR) PFS 65-74 years: 0.52 (NR) PFS <65 years: 0.65 (NR)
IMpower 130^[26]^	lll First line	Untreated nonsquamous	Platinum-based chemotherapy±Atezolizumab	Primary: OS, PFS Secondary: ORR, DOR Safety	n=723 ≥65 years: 50%	OS ≥65 years: 0.78 (0.58-1.05) OS <65 years: 0.79 (0.58-1.08) PFS ≥65 years: 0.64 (0.50-0.82) PFS <65 years: 0.64 (0.50-0.82)
IMpower 131^[27]^	lll First line	Untreated squamous	Platinum-based chemotherapy±Atezolizumab	Primary: OS, PFS Secondary: PFS, OS in subgroups Safety	n=1021 Median age 65 years ≥65 years: 52%	PFS ≥75 years: 0.51 (0.30-0.84) PFS 65-74 years: 0.66 (0.51-0.87) PFS <65 years: 0.77 (0.61-0.99)
CheckMate 227^[30]^	lll First line	Part 1A: PD-L1 TPS≥1% Part 1B: PD-L1 TPS <1%	Nivolumab+Ipilimumab vs Platinum-based chemotherapy	Primary: Part 1A: OS Part 1B: PFS	n=1189 Median age 64 years ≥65 years: 49%	OS ≥75 years: 0.85 (0.56-1.28) OS 65-74 years: 0.75 (0.61-0.92) OS <65 years: 0.71 (0.59-0.85)
CheckMate 9LA^[31]^	lll First line	Untreated EGFR/ALK negative	2 cycles Platinum-based chemotherapy+ Nivolumab+Ipilimumab vs Platinum-based chemotherapy	Primary: OS Secondary: PFS, ORR	n=719 Median age 65 years ≥65 years: 51%	OS ≥75 years: 1.21 (0.69-2.12) OS 65-74 years: 0.62 (0.46-0.85) OS <65 years: 0.61 (0.47-0.80)

EGFR: epidermal growth factor receptor; ALK: anaplastic lymphoma kinase; OS: overall survival; PFS: progression-free survival; ORR: objective response rate; DOR: duration of response; ITT: intention-to-treat; NR: not reached; NSCLC: non-small cell lung cancer; TPS: tumor proportion score.

### 2.4 免疫联合放疗

肿瘤放射治疗是局部治疗的手段之一，不仅可以直接杀伤肿瘤，还可以在与免疫治疗联合使用时，增强肿瘤免疫原性、产生远隔效应、改变肿瘤微环境，从而增强免疫系统杀伤肿瘤的作用。免疫治疗则可以通过重塑肿瘤血管、减弱放射抗性和促进远隔效应发生起到对放疗的增敏作用^[32]^。PACIFIC试验^[33]^使局部晚期的NSCLC治疗模式发生转变，评估接受同步放化疗后续贯免疫治疗或安慰剂的疗效，结果显示续贯免疫治疗组的中位PFS（16.9 vs 5.6个月）和中位OS（47.5 vs 29.1个月）均较安慰组延长，说明同步放化疗联合免疫治疗有持久的临床获益，>65岁患者疗效与年轻患者无差异（HR=1.30, 95%CI: 1.06-1.59）。LUN14-179试验^[34]^也得出了同样的结论。对于IV期NSCLC，PEMBRO-RT试验探究免疫单药联合放疗的疗效与安全性，发现联合治疗组中位OS、中位PFS、客观缓解率（objective response rate, ORR）均优于免疫单药组，且>65岁患者与年轻患者无疗效差异（HR=2.24, 95%CI: 1.03-4.86）^[35]^。综上，ICIs联合放疗治疗可以改善NSCLC预后，且老年患者与年轻患者疗效无明显差异。

### 2.5 免疫联合抗血管治疗

肿瘤局部血管的异常会形成乏氧的免疫抑制微环境，抗血管生成治疗可以促进肿瘤抗原的呈递及免疫应答细胞的浸润，并减少免疫抑制细胞发挥的积累，使肿瘤血管正常化，并且可以促进高内皮性小静脉（high endothelial venules, HEV）的生成，促进B、T细胞活化，从而触发进一步的抗肿瘤效应^[36]^。免疫治疗则通过刺激分泌干扰素-γ（interferon-γ, INF-γ），减轻局部缺氧，促进肿瘤血管正常化，二者是一个正反馈作用^[37]^。IMpower 150研究^[25]^中四药联合组和化疗联合抗血管生成组患者的中位PFS（8.3 vs 6.8个月）和中位OS（19.2 vs 14.7个月）显著延长，在AEs上两组总体上相似，说明抗血管生成药物与ICIs存在协同作用。JVDF试验^[38]^旨在评估雷莫芦单抗联合帕博利珠单抗治疗晚期实体瘤的疗效，其中NSCLC患者的ORR为42.3%，疾病控制率（disease control rate, DCR）为84.6%，中位PFS为9.3个月且耐受性良好。一项研究^[39]^发现帕博利珠单抗联合安罗替尼对比帕博利珠单抗单药治疗既往治疗失败的EGFR突变NSCLC，中位PFS及中位OS均明显获益，且在>65岁患者和年轻患者中无统计学差异。免疫联合抗血管治疗可显著改善晚期NSCLC患者的预后，且安全性可耐受。遗憾的是，现有的试验中较少对老年患者的生存改善进行研究，因此该方案对于老年患者的实际获益情况有待更多临床试验补充。

## 3 老年晚期NSCLC患者ICIs治疗的不良反应

ICIs治疗是癌症治疗领域的重大突破，改善了NSCLC和其他晚期实体瘤患者的预后。但它会导致患者出现irAEs，程度从轻微到严重不等，最常受累的有内分泌系统、皮肤、结肠、肺和肝脏，最常见的症状是腹泻、瘙痒、皮疹和疲劳等，irAEs可以发生在治疗过程中的任何时间，甚至停药后的几个月^[40]^。

KEYNOTE-010、-024、-042研究的汇总分析^[17]^有149例≥75岁老年患者接受免疫治疗，他们与<75岁人群的irAEs的发生率相似，分别为24.8%和25.0%，≥3级irAEs的发生率分别为9.4%和7.1%。一项来自法国的研究^[41]^发现，与年轻患者相比，ICIs单药治疗后出现≥2级irAEs的发生率在70岁以上患者中更高（33% vs 25%），但严重程度各组无统计学差异。另一项回顾性研究^[42]^包含527例NSCLC患者，ICIs单药治疗irAEs的发生率在不同年龄组间没有差异，但75岁以上患者因irAEs中断治疗更常见。针对免疫联合化疗，一项回顾性研究^[43]^发现，3-5级肺炎的发生率在使用免疫联合化疗的老年患者中显著升高（16.0% vs 2.1%）。KEYNOTE-189与KEYNOTE-407等针对免疫联合化疗的临床试验^[23,24]^同样表明联合治疗组比单纯化疗组irAEs发生率更高，但总体AEs发生率相近。

关于老年晚期NSCLC患者出现irAEs的研究较少，综上所述，使用ICIs单药治疗在不同年龄段的患者中具有相似的irAEs发生率，但是对于≥75岁或者一般状况较差的老年人来说，irAEs的影响仍不可忽略。免疫联合化疗对比单纯化疗导致irAEs的发生率高，老年患者似乎更容易发生更高级的irAEs，同时考虑到联合用药带来的影响，在对老年晚期NSCLC患者进行治疗前需要更加谨慎地全面评估，选择更适合其治疗的方案。

## 4 老年晚期NSCLC治疗展望

综上，老年患者免疫治疗的获益与安全性与年轻患者无明显差异。免疫联合抗血管治疗及放疗可以改善预后且相对安全，然而没有专门针对老年患者进行研究。对于老年患者，除了年龄，ECOG PS评分、合并症、其他用药情况都会影响预后。老年患者异质性大，单一ECOG PS评分不足以完全反映其整体状况。因此需要进行综合评估，以平衡个体患者治疗的安全性和有效性^[44]^。一项针对筛查工具的研究^[45]^提出老年-8问卷（Geriatric-8, G8）敏感性强，有助于识别易受伤害或虚弱的患者，弱势老年人调查-13问卷（Vulnerable Elders Survey-13, VES-13）特异度高，两者结合可以很好地满足老年综合评估的要求。癌症及衰老研究组（Cancer and Aging Research Group, CARG）化疗风险评估量表可评估化疗风险，预测老年肺癌患者的化疗耐受性，有助于制定个体化的治疗方案^[44]^。除此之外，美国临床肿瘤协会（America Society of Clinical Oncology, ASCO）的指南^[46]^中，建议老年肿瘤患者以老年综合评估（comprehensive geriatric assessment, CGA）取代或补充传统的肿瘤学评估指标，从而更好地识别化疗毒性、死亡率、功能下降和其他不良后果的风险。无论是何种评估工具，都说明老年患者能否从免疫治疗中获益，年龄不是根本因素，需要多维度综合评估，指导个性化的老年肿瘤治疗方案。

免疫治疗和化疗是两种癌症领域常见的治疗方式，主要通过以下机制发挥协同作用：（1）化疗诱导肿瘤细胞免疫原性死亡（immunogenic cell death, ICD）^[22]^；（2）化疗增强肿瘤细胞的免疫原性^[22]^；（3）化疗耗竭免疫抑制细胞^[22]^；（4）免疫治疗可以增强和恢复化疗敏感性等^[47]^。对于晚期肺癌患者，为得到更好的疗效，常采用二者联合治疗的方式。但是老年患者由于自身特点，对标准剂量化疗相关毒性的耐受能力较差，不良反应发生风险高，所以可以尝试免疫联合低强度化疗的方案。

有研究^[48]^对经治疗后病情进展的老年晚期NSCLC患者采用了替雷利珠单抗联合低剂量白蛋白结合型紫杉醇的方案，截至观察日期，PFS与OS分别为9.5和16.5个月，58.6%患者发生≥1次治疗相关AEs，10.3%患者发生3级AEs，表明老年晚期NSCLC患者对该治疗方案的耐受性良好且有效。另一项病例报告^[49]^对1例经多线治疗后病情进展的老年晚期肺腺癌患者采用卡瑞利珠单抗联合低剂量地西他滨的方案，使用2个周期评估时发现患者转移病灶缩小，疼痛症状缓解，遗憾的是治疗7个周期后患者病情进展，但仍可以看出这种治疗方案的潜力。目前对于低强度化疗并没有标准的定义，根据既往有限相关研究^[50]^及临床经验将标准化疗剂量减少20%及以上或将标准双药化疗中的一种化疗药物减除暂时认为是低强度。相较于标准剂量化疗，低强度化疗不仅可以通过减少肿瘤负荷和增加免疫原性来提高免疫治疗的效果，还能够减轻患者的毒副作用，提高老年患者的治疗耐受性，从而为免疫治疗提供更为良好的治疗基础，是一种值得尝试的治疗方案，有为老年晚期NSCLC患者带来更好疗效同时保证安全的潜力。老年患者常被各种临床试验排除在外，但随着全球老龄化的来临，我们期望开展更多针对老年患者的临床试验，以期为这部分占比较大的患者在保证安全性的前提下提供合理有效的治疗方案。

## 5 总结

肺癌居我国男、女性癌症发病和死亡首位，NSCLC患者中老年占比较高。≥65岁的老年晚期NSCLC患者ICIs单药或联合化疗的疗效优于单纯化疗，且与年轻患者无显著的疗效差异。而在≥75岁患者中，由于数据有限，免疫治疗获益并不明显。除此之外，免疫联合放疗或抗血管治疗也显著改善患者预后，为老年晚期NSCLC患者带来新的希望。免疫治疗与化疗相互协同，但部分老年人对于标准化疗耐受性差，免疫联合低强度化疗具有一定的潜力。

老年癌症患者的比例随着人口的老龄化逐步增加，应有意识增加老年患者入组ICIs临床试验的人数，同时也应综合多维度评估，探索个体化治疗方案，为优化老年癌症患者治疗方案提供更科学的依据。
